# Study on Possible Application of Rubber Granulate from the Recycled Tires as an Elastic Cover of Prototype Rail Dampers, with a Focus on Their Operational Durability

**DOI:** 10.3390/ma14195711

**Published:** 2021-09-30

**Authors:** Cezary Kraśkiewicz, Bogumiła Chmielewska, Artur Zbiciak, Anna Al Sabouni-Zawadzka

**Affiliations:** 1 Institute of Roads and Bridges, Faculty of Civil Engineering, Warsaw University of Technology, Al. Armii Ludowej 16, 00-637 Warsaw, Poland; a.zbiciak@il.pw.edu.pl; 2 Institute of Building Engineering, Faculty of Civil Engineering, Warsaw University of Technology, Al. Armii Ludowej 16, 00-637 Warsaw, Poland; b.chmielewska@il.pw.edu.pl (B.C.); a.sabouni@il.pw.edu.pl (A.A.S.-Z.)

**Keywords:** rail dampers, noise reduction, track structure, elastic characteristics, operational durability

## Abstract

This study is an attempt to investigate possible applications of rubber granulate SBR (styrene-butadiene rubber) produced from recycled waste tires as an elastic cover for prototype rail dampers, which are aimed at reducing the level of railway noise emitted in the environment. The authors present laboratory procedures and discuss the results of several experimental tests performed on seven different SBR materials with the following densities: 1100, 1050, 1000, 850, 750, 700 and 650 kg/m^3^. It is proven that rubber granulate SBR produced from recycled waste tires, can be used as an elastic cover in steel inserts in rail dampers, provided that the material density is not lower than 1000 kg/m^3^. In the conducted tests, samples of the materials with high densities exhibited good static and dynamic elastic characteristics and had sufficient operational durability.

## 1. Introduction

Increased train velocity leads to shorter travel times, but at the same time has significant negative effects, such as increased noise emission, which affect both people and the environment. In order to minimize this negative phenomenon various methods are used which are aimed at reducing the level of railway noise. One of the commonly used methods is the application of noise barriers along railways lines. Such traditional solutions, however, may not always be used, for technical (location, required dimensions), economical or aesthetic reasons. Moreover, in some cases they do not lead to satisfactory results. The International Union of Railways published a state of the art report [1] on noise mitigation options, where the three main solutions discussed and compared to traditional, widely used noise barriers were rail dampers, acoustic rail grinding and low height noise barriers. The traditional solutions are not always effective or possible to apply. Their effectiveness, however, may be enhanced by using additional innovative solutions, such as the rail dampers discussed in this paper. The proposed solution is the subject of a research project, “Innovative solutions for the protection of people and the environment against rail traffic noise”, or InRaNos, realized by the Faculty of Civil Engineering at the Warsaw University of Technology (WUT). Prototype rail dampers can be used as elements that protect people and the surrounding environment against the negative influence of noise induced by railway traffic. Such solutions have never been used in Poland before, but based on foreign studies [1] where rail dampers with elastic polyurethane covers were primarily implemented, they exhibit great potential for increasing the effectiveness of the noise level suppression compared to traditional methods.

**Figure 1:**
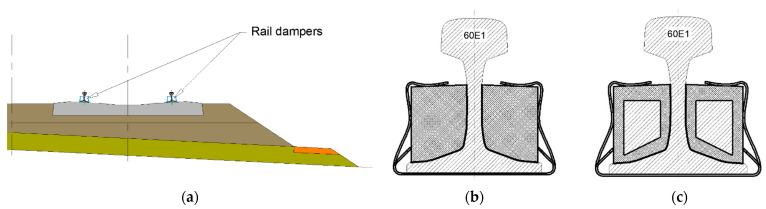
Prototype rail dampers: (**a**) Schematic location within the ballasted track structure; (**b**) Cross-section of a static damper; (**c**) Cross-section of a dynamic damper with a steel insert and an elastic cover based on rubber granulate SBR.

Rail dampers are elements that are fixed to both sides of the rail (the rail web and/or rail base, and in some cases even to the bottom part of the rail head); they are attached to the rail by gluing or using additional elastic clamping elements. Rail dampers are usually placed at equal intervals along the rail (Figure 1a) in sections between rail fastenings. They are sometimes also referred to as “absorbers”.

The main function of rail dampers is to accelerate the decay of vibrations induced in the rail (the TDR, or Track Decay Rate [2]) and to thereby reduce the level of noise emitted into the environment. Two types of rail dampers may be distinguished:

- Dampers with an elastic cover that either fully or partially fills the rail chambers and, in some cases, also surrounds the surface of the rail base. The rail cover exhibits the same characteristics of vibration as the rail, so it does not change the general dynamic characteristics of the rail system. This type of damper will be referred to as “static dampers” or “dampers with cover” (Figure 1b);
- Dampers with an element or a group of elements with a certain mass that are distributed periodically along the rail chambers and fixed to the rail using an elastic elastomeric layer (usually polyurethane resin). The elastically attached damping mass (usually a steel insert) is excited by the moving trains; due to the elastic layer, the induced vibrations can undergo a phase shift compared to the rail vibrations. This phenomenon changes the general dynamic characteristics of the rail, and thereby suppresses the acoustic wave emitted by the rail, especially its web. This version of rail damper will be referred to as “mass dampers” or “dynamic dampers” (Figure 1c).

The effectiveness of rail dampers depends on various aspects such as the fastening method, type of elastomeric material, shape and material of the inserts, etc. It can be verified by direct measurements or determined based on dynamic characteristics analysis, or TDR [3]. The studies conducted by Sieglitz et al. [4] proved that the effectiveness of rail dampers ranges from 2 dB to 6 dB, depending on the type and velocity of the trains. This factor has a significant influence for velocities of 40 ÷ 80 km/h. Above this range, the damping effectiveness is constant and independent from the train velocity. Moreover, the studies revealed that the roughness of the rails and wheel running surfaces does not affect the effectiveness of rail dampers.

Many researchers focus on searching for solutions to enhance the isolation properties of UBMs and other resilient elements used in track structures. Sol-Sánchez et al. [5,6,7] presented analyses of rail pads under sleeper pads (USPs) and under ballast mats (UBMs) produced from recycled end-of-life tires. The proposed solutions were used to diminish global stiffness of the ballasted track system and to suppress vibration. Onorii et al. [8] determined static and dynamic characteristics of the track with rubber-based UBMs manufactured from recycled tires. They showed a positive effect of the tested vibration isolators on eigenfrequencies of the track structure and justified that the deflections of the track structure should be considered when determining material characteristics of the mats.

The recycled rubber discussed in the above mentioned papers [5,6,7,8] is often applied to improve the behavior of elements in various areas of civil engineering not necessarily related to railways. Gardziejczyk et al. [9] showed that the addition of rubber granulate can positively affect the viscoelastic properties of stone mastic asphalt, reducing tire/road noise. Contreras-Marín et al. [10] considered the use of granulated rubber tire waste as lightweight backfill material for retaining walls. Klajn et al. [11] analyzed SBR vulcanizates filled with modified ground tire rubber (GTR). They proved that the addition of modified GTR increases the stiffness of the vulcanizates. Kim et al. [12] determined the mechanical and dynamic behavior of an elastic rubber layer in synthetic sports surfaces produced using waste tire chips containing SBR. They proved that the recycled material can be used to enhance the mechanical properties of surfaces.

There are many methods for assessing the performance of rail dampers. A series of papers [13,14,15] focuses on a Franco-German research project, STARDAMP, which was aimed at supporting the transfer from research and development of rail and wheel dampers to their application. Betgen et al. [15] described a software tool that can be used to predict the effectiveness of wheel and rail dampers. In [16] the authors proposed a method for the prediction of TDR which is based on the finite element modelling (FEM).

Squicciarini et al. [17], on the other hand, focused on laboratory tests for evaluating the performance of rail dampers. They measured vertical and lateral decay rates on a free rail equipped with dampers. Haladin et al. [18] stated that the rail track vibration damping level is a crucial property while determining the proportion of rail track influence in the total rail traffic noise and vibration levels. Qian et al. [19] studied the effects of a rail vibration absorber on suppressing short pitch rail corrugation. They performed field tests and additionally applying two FE models of a wheel–rail system and a wheel–rail-absorber system. Michalczyk et al. [20] introduced a concept and presented preliminary results of numerical FEM analyses of changes in the rail track dynamic characteristics. The conducted research revealed a dependency between the increase of the railway track mass and its dynamic characteristics, and thus, the noise emission. Zoontjens et al. [21] studied the rolling noise emissions caused by trains running on the Perth electrified passenger network. They compared STARDAMP model results with field test results for the ballasted and slab track, with and without rail dampers. They proved that the noise induced by the train network can be effectively attenuated with the use of rail dampers.

The present paper is a study on possible application of rubber granulate from re-cycled waste tires as an elastic cover for prototype rail dampers, which are aimed at reducing the level of railway noise emitted to the environment. The authors discuss laboratory procedures and present results of experimental tests performed on seven different SBR materials with the following densities: 1100, 1050, 1000, 850, 750, 700 and 650 kg/m^3^. It is proved that the rubber granulate SBR produced from recycled waste tires can be used as an elastic cover for the steel insert. The proposed prototype rail dampers exhibit sufficient operational durability within the density range of 1000 ÷ 1100 kg/m^3^.

## 2. Experimental Identification of Static and Dynamic Elastic Characteristics

### 2.1. Standards and Test Procedures

Due to the fact that there is no valid European standard that could be used for determination of the static and dynamic characteristics of the elastic rail damper covers, the authors decided to use the procedures described in the German standard DIN 45673-7 [22]. It must be mentioned that according to this standard the laboratory tests should be performed with the use of a flat steel plate in order to ensure the proper simulation of continuous contact between the side and bottom surface of the damper elastic cover and the web and base of the Vignole rail (see Figure 1c) in the ballasted track system (see Figure 1a). Varying ranges of assessed loads in the static tests and a three-step initial static load in the dynamic tests result from the varying clamping force of the designed prototype clamping elements made of spring steel. Moreover, such an assumption takes into account the possibility of a change in the initial clamping force if the damper were to be reinstalled periodically due to maintenance work.

Within the wide range of performed laboratory tests not only static and dynamic characteristics were identified, but also tensile strength and elongation at break according to ISO 1798 [23]; these are typical parameters that characterize elastomeric materials.

In the presented research it was assumed that the tested rail dampers should have a designated service life similar to other elements of the track structure, mainly rails. In Poland, the minimum service life of rails is 20ߝ25 years, depending on the rail type (60E1/49E1) and service loads (train velocity, axial loading and transport intensity, ca. 500 Tg for 60E1 and 250 Tg for 49E1) [24].

### 2.2. Description of the Samples

**Figure 2:**
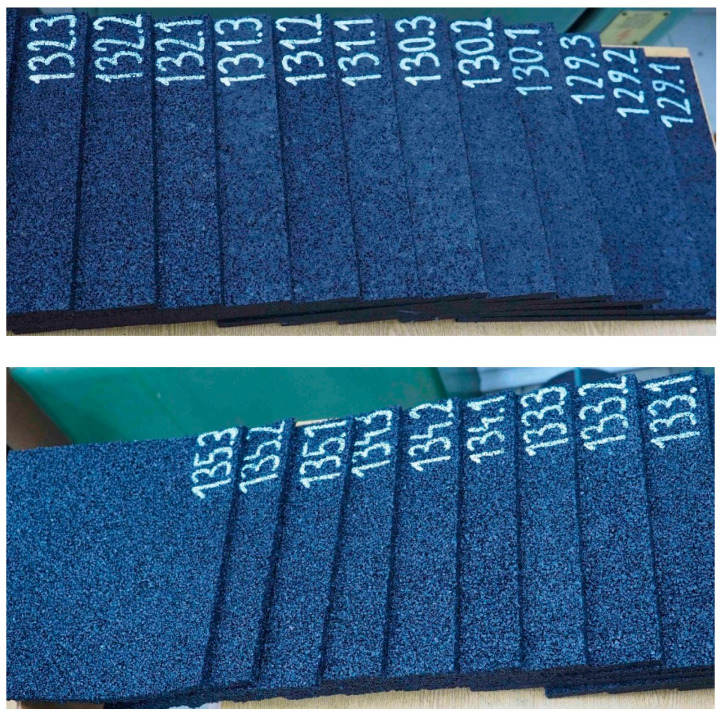
SBR samples with visible porosity increase for the materials with lower densities (higher numbers).

Within the tests, samples of seven materials intended to be used as elastic covers for rail dampers were investigated. The tested samples were numbered 129, 130, 131, 132, 133, 134 and 135, and were made of the rubber granulate SBR with a thickness of 10 mm and corresponding densities of 1100, 1050, 1000, 850, 750, 700 and 650 kg/m^3^, respectively Figure 2 presents a picture of the samples, where an increased porosity of the material with lower densities can be noticed. It was assumed that the dimension of 10 mm is a minimum value for the elastic cover of the steel insert that is located inside the rail damper (see Figure 1c).

The samples were produced from elastomeric materials based on the rubber granulated from the recycled waste tires. Such materials should not lose their structural continuity under tension or compression; they regain their initial shape after load removal and exhibit significant elongation while in tension.

Sheets of elastic rubber-based material with various densities and equal thickness of 10 mm were prepared in the elastomer factory. Afterwards, the oar-shaped samples with the dimensions specified in Section 2.5 were cut using water jet cutting. The chosen method makes it possible to obtain accuracy to dimensions of 0.1 mm, ensure even edges and cut very thick materials. It does not induce thermal loads and is 100% safe. All tests of tensile strength and elongation at break were performed on six samples with equal dimensions and the same thickness of 10 mm for each of the analysed densities.

### 2.3. Identification of Static Elastic Properties

**Figure 3:**
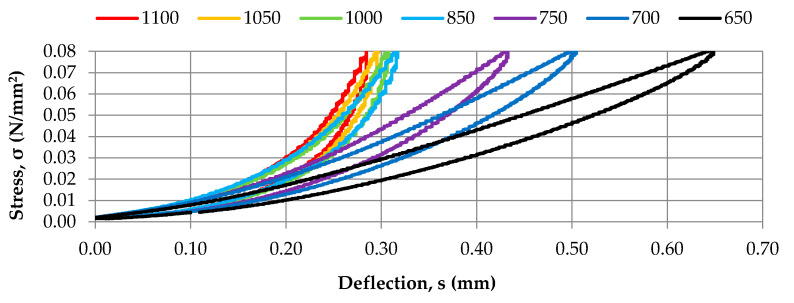
Diagrams of static characteristics of the elastic cover of prototype rail dampers obtained for seven densities of SBR granulate (650–1100 kg/m^3^).

Static tests were conducted on samples with the dimensions of 300 mm × 300 mm × 10 mm, three samples for each analyzed density of SBR granulate. Identification of static bedding moduli according to DIN 45673-7 [22] was performed for the range of the applied loads of 0–0.080 N/mm^2^ and the following ranges of assessed loads: 0.005–0.020 N/mm^2^; 0.010–0.040 N/mm^2^; 0.020–0.050 N/mm^2^; 0.020–0.070 N/mm^2^. Diagrams of the static elastic characteristics (stress-deflection) obtained for tested samples of the elastic covering of rail dampers are presented in Figure 3 and the determined values of static bedding moduli are gathered in Table 1. The static bedding moduli (Table 1) were determined based on the stress-deflection diagram (Figure 3) using the secant method, in accordance with the assessed load range.

### 2.4. Identification of Dynamic Elastic Properties

Dynamic properties of materials are often represented by the damping ratio and shear modulus, especially in regard to subgrade soils [25]. In the case of elastic materials used in track structures, however, identification of dynamic characteristics of the material is performed in accordance with the standards concerning particular elements of the structure: rail pads (EN 13146-9), USPs (EN 16730), and UBMs (EN 17282), where the dynamic bedding modulus is the most crucial parameter. All these standards concern determination of static and dynamic characteristics of vibration isolators submitted to cyclic compression. Examples of such tests are presented in several previous works of the authors [26,27,28]. Analogical assumption was made in the static and dynamic tests performed on the elastic cover of the steel insert of the analysed rail dampers, which was pressed to the rail by steel clamps.

Dynamic tests were conducted on samples with the dimensions of 300 × 300 × 10 mm, three samples for each analyzed density of SBR granulate. Identification of dynamic bedding moduli according to DIN 45673-7 [22] was performed with the three-step initial static loading of 0.020 N/mm^2^, 0.030 N/mm^2^, 0.040 N/mm^2^ and the load frequencies of 5 Hz, 10 Hz, 15 Hz and 20 Hz. Dynamic excitation with different frequencies was realized using the displacement controlled pulsator and with the constant particle velocity of 100 dB (the reference value according to EN ISO 10846-2 [29] is 5 × 10^–8^ m/s).

Additionally, going beyond the standard requirements of DIN 45673-7 [22], the authors tested dynamic bedding moduli with the force controlled pulsator for the range of the applied and assessed loads of 0.010–0.040 N/mm^2^ and the frequencies of 1, 2, 3, 5, 10, 15 and 20 Hz.

The selected range of frequencies resulted from the limitations of the universal testing machine, Instron 8802, that was used for the tests. The testing procedure described in EN ISO 10846-2 [29] allows for higher frequencies. However, based on theory and experimental results [30], the biggest increase in the dynamic bedding modulus occurs for frequencies below 20 Hz, and above this value it grows asymptotically to the constant value. The excitation frequency range depends on the train velocity, but also on other factors, such as distance between train carriages and their axes, distance between sleepers, rail corrugation and many others.

**Figure 4:**
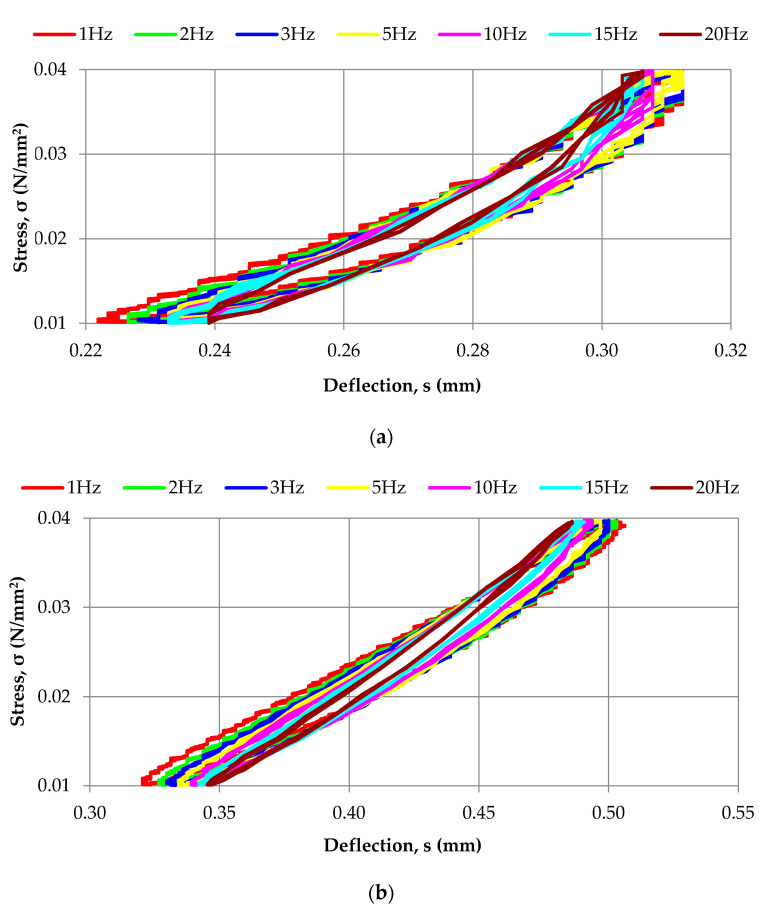
Diagrams of dynamic characteristics of the elastic cover of prototype rail dampers obtained for two extreme densities of SBR granulate: (**a**) 1100 kg/m^3^; (**b**) 650 kg/m^3^.

Examples of the dynamic elastic characteristics obtained for two materials with extreme densities (1100 kg/m^3^ and 650 kg/m^3^) are shown in Figure 4, and the dynamic bedding moduli determined for all analyzed densities are presented in Table 2. It can be noticed that the slope of the tangent line in the stress-deflection characteristics increases with the increase of the load frequency and, at the same time, the area of the hysteresis loop decreases (see Figure 4). This results in smaller damping effectiveness of the elastic cover under higher load frequencies, and is noticeable with larger values of the dynamic bedding moduli obtained for such frequencies (see Table 2). It is a typical phenomenon observed in elastomers, which exhibit simultaneously viscous and elastic behaviour and stiffen when the load frequency increases.

### 2.5. Tensile Strength and Elongation at Break

**Figure 5:**
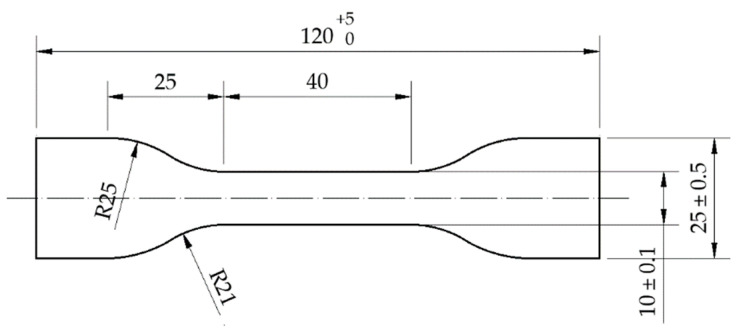
Sample dimensions in mm.

**Figure 6:**
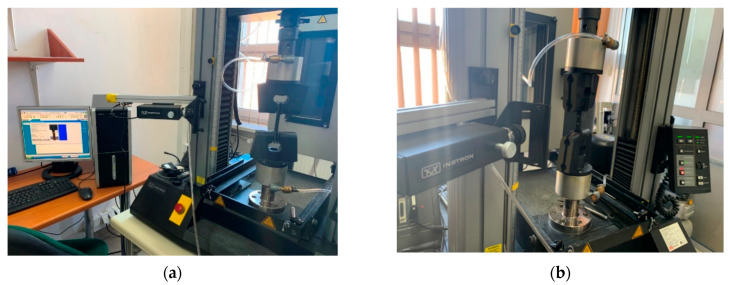
Tensile test of the elastomeric materials: (**a**) test stand; (**b**) sample with visible marks and video extensometer before the test.

The tests were conducted on seven previously described elastomeric materials made of SBR granulate with various densities. Oar-shaped samples with the dimensions of 120 × 25 × 10 mm (Figure 5) were submitted to tension with the velocity of 500 mm/min until break according to EN ISO 1798 [23]. The elongation was controlled with a video extensometer (Figure 6).

**Figure 7:**
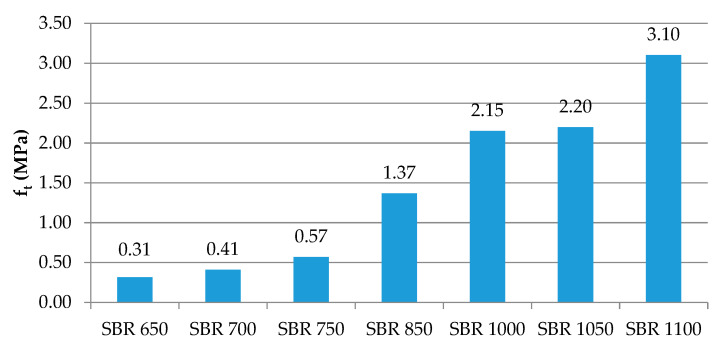
Tensile strength depending on the elastomeric material density.

**Figure 8:**
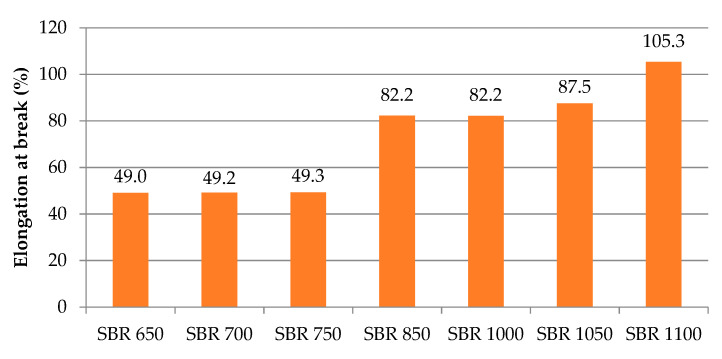
Elongation at break depending on the elastomeric material density.

The tests revealed that together with an increase of the material density from 650 to 1100 kg/m^3^, the tensile strength increased from 0.31 to 3.10 MPa (Figure 7), with a coefficient of variation of 2.6% to ca. 12%. The elongation at break also grows for higher densities, that is, from ca. 49% to ca. 105% (Figure 8), with the coefficient of variation ranging from 3.6% to 11.6%.

### 2.6. Conclusions from the Experimental Identification of Elastic Properties

Results of the performed tests lead to the following conclusions:

- Variations of the static and dynamic elastic properties are small in the case of SBR samples with densities of 1000 kg/m^3^ or higher (SBR 1000, SBR 1050 and SBR 1100);
- Variations of the static and dynamic elastic properties are significant in the case of SBR samples with densities below 1000 kg/m^3^ (SBR 850, SBR 750, SBR 700 and SBR 650);
- Tensile strength and elongation at break decrease significantly in the case of SBR samples with densities below 1000 kg/m^3^ (SBR 850, SBR 750, SBR 700 and SBR 650).

## 3. Experimental Verification of the Operational Durability

### 3.1. Adopted Assumptions

The operational durability of elastomeric materials, including their resistance to severe environmental conditions and chemicals coming from the operating trains (e.g., oils), is a very important property that influences the durability of the whole track system and the effectiveness of the protection against railway noise. Elements of the railway infrastructure must fulfill high requirements for operational durability, as they affect railway traffic safety. They cannot undergo any damage during their operation, and the variation of the previously identified elastic properties must be low. The elastic cover cannot undergo significant expansion (swelling) in contact with oils, because this could lead to detachment of the rail damper from the rail, destruction of clamping elements or lower effectiveness of noise damping.

Additionally, rail dampers should exhibit sufficient resistance against damage to their elastic cover caused by impacts of sharp edged ballast grains and other elements including iron ores and coal waste that can drop and spread out along the track and thus reduce damper performance [31]. Such impacts can be caused by the aerodynamic effect that occurs when the train moves with high velocity and ballast grains are rapidly raised, or can occur during construction or maintenance work.

Apart from oil leakage and impacts from ballast grains, mining products including iron ores and coal waste can drop and spread out along the track, which can reduce damper performance.

In order to verify operational durability, the authors investigated the influence of selected environmental conditions (high and low temperature and UV radiation) and chemicals that could come from the operating trains (e.g., oils) on the physical properties of elastomeric materials, based on SBR granulate from the recycled tires. Moreover, resistance testing of the elastic cover of rail dampers in contact with the geometric ballast plate (GBP) was performed, in order to make sure that no damage, including permanent deformation of the elastic cover, could occur.

Apart from the performed tests, the resistance of rail dampers to fire should be tested. Rail dampers are used from time to time in railway line sections located in tunnels and underground stations, where the air circulation is limited. The laboratory at WUT, however, is not adapted for fire resistance tests; this is the reason why such tests are not included in the paper. As far as the fire requirements are concerned, in the German railway (DB), for example, according to DBS 918 290 [32] rail dampers applied in tunnels should be certified for flammability class A (according to DIN 4102-1 [33] or EN 13501-1 [34]). For other elastomeric materials used, for example, as UBMs, railway infrastructure managers formulate lower requirements: in German, Swiss and Italian railways they should be designed for class B2. These lower requirements result from the location of UBMs, which are further from potential fire sources than rail dampers.

### 3.2. Influence of High Temperature and UV Radiation on Tensile Strength and Elongation at Break

**Figure 9:**
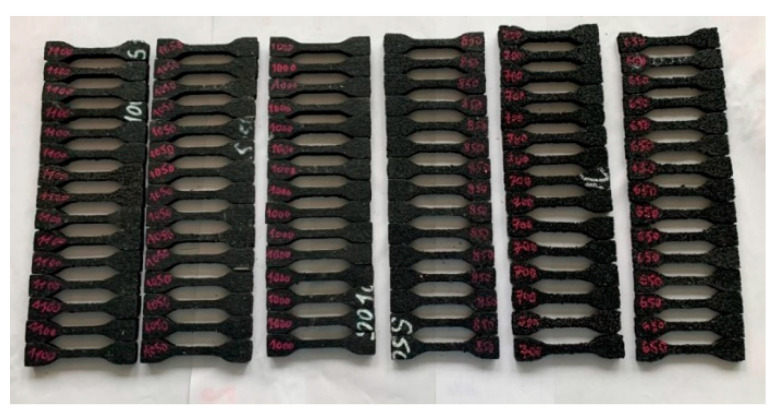
Samples of the elastomeric material with various densities prepared for ageing tests (high temperature and UV radiation).

Samples of six elastomeric materials (Figure 9) were submitted to accelerated ageing testing according to EN ISO 2440 [35]. One of the previously described samples, SBR 750, was not tested because of the fact that it exhibited very similar behavior to SBR 700 (see Table 1 and Table 2 and Figure 7 and Figure 8), and due to the limited space in the climatic chamber.

Two properties were tested, tensile strength and elongation at break, as well as their changes in comparison with the samples kept in standard conditions (see Section 2.5).

The following testing conditions were assumed:

- Oar-shaped samples with the dimensions of 120 × 25 × 10 mm;
- Number of samples: 5;
- Test temperature: +70 °C;
- UV radiation corresponding to the solar radiation;
- Testing period: 168 h (7 days).

**Figure 10:**
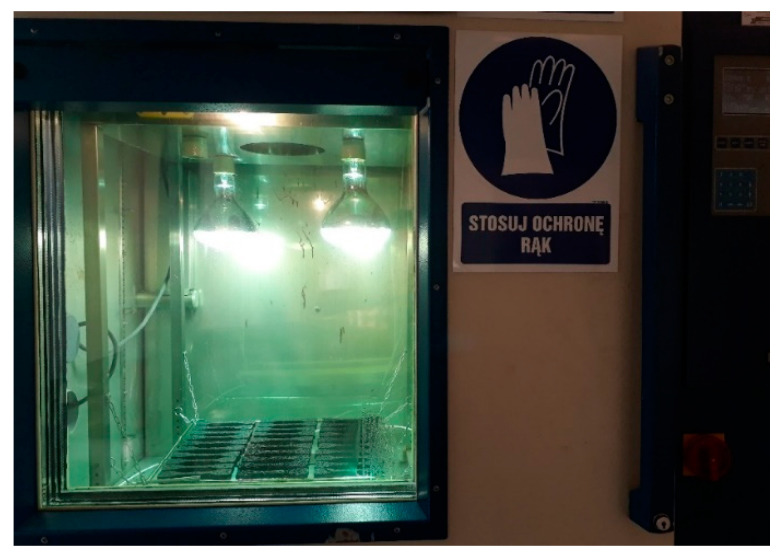
Samples of the elastomeric material in the climatic chamber Feutron, simulating high temperature and solar radiation.

The tests simulating solar radiation were conducted in laboratory conditions, in the climatic chamber Feutron (type 3523/15, Feutron Klimasimulation GmbH, Langenwetzendorf, Germany, 2000) (Figure 10) equipped with a test stand constructed in accordance with the requirements of EN ISO 4892-3 [36]. During the tests, the samples were submitted to the radiation UVA 315–400 nm (13.5 W) and UVB 280–315 nm (3 W).

**Figure 11:**
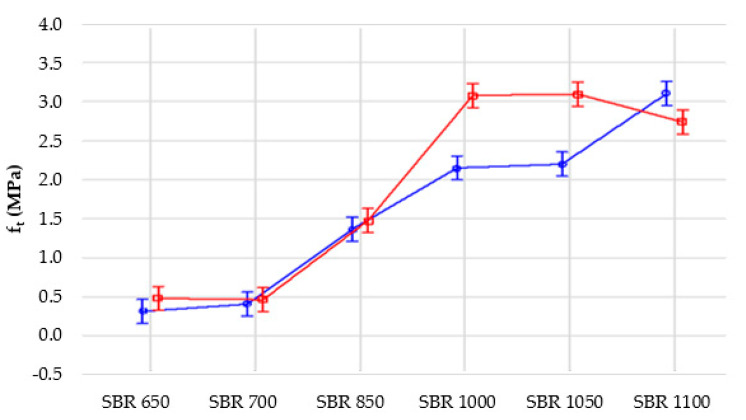
Influence of density on tensile strength of the samples for two analyzed conditions: blue line—1 (normal conditions), red line—2 (ageing conditions).

**Figure 12:**
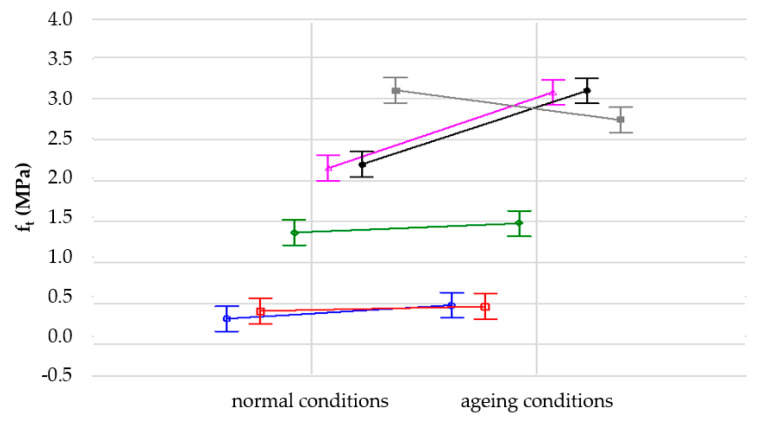
Influence of conditioning on tensile strength of the analyzed elastomeric materials with various densities: blue line—SBR 650, red line—SBR 700, green line—SBR 850, purple line—SBR 1000, black line—SBR 1050, grey line—SBR 1100.

In order to analyze the influence of samples conditioning (1—normal conditions “N”, 2—ageing conditions “UV + T”) on their tensile strength, a variance analysis was performed. It was proved that each of the analyzed factors (normal conditions, ageing and their interaction) significantly affects the mean values compared to the sampling error (Table 3, Figure 11 and Figure 12). This influence is most noticeable in the case of material density; here, it is more significant than the interaction between density and test conditions.

**Figure 13:**
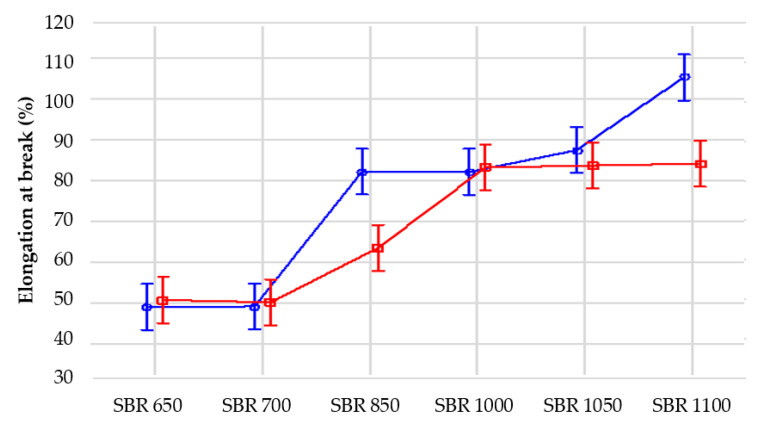
Influence of density on elongation at break of the samples for two analyzed conditions: blue line—1 (normal conditions), red line—2 (ageing conditions).

**Figure 14:**
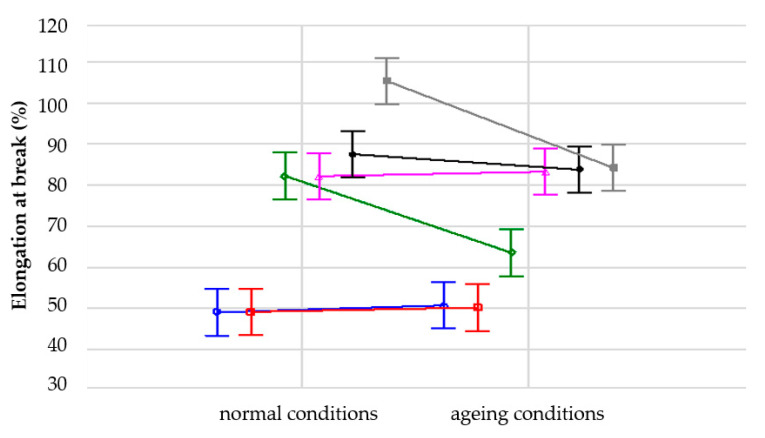
Influence of conditioning on elongation at break of the analyzed elastomeric materials with various densities: blue line—SBR 650, red line—SBR 700, green line—SBR 850, purple line—SBR 1000, black line—SBR 1050, grey line—SBR 1100.

Similar analysis was performed for the elongation at break measurements. It was proven that each of the analyzed factors significantly affects the mean values compared to the sampling error (Table 4, Figure 13 and Figure 14). Furthermore, in this case this influence is most noticeable in the case of material density, more significant here than in the interaction between density and test conditions.

It should be mentioned that sheets of elastic rubber-based material with various densities and equal thickness of 10 mm were prepared in a factory where an automated production line is used for the manufacturing of materials based on rubber granulate and fibres. The manufacturer uses a quality control system which is consistent with ISO 9001, and some additional inner procedures for production quality control. All produced sheets were checked and verified based on such parameters as hardness, thickness and density. The material of the sheets was manufactured from small granules of rubber with a diameter of a few millimetres coming from deconstructed waste tires. Damage to the tires had no influence on the properties of the new material, as they were crumbled mechanically and carefully mixed. All sheets with various densities were produced on the same production line and based on the same batch of rubber granulate and polyurethane adhesive.

However, it should be noticed that the SBR-based samples are composites which, apart from different densities, may differ in the amount of polyurethane adhesive used to join rubber granules. The amount of adhesive was selected for each density separately, within the prototypical recipe, and this could have resulted in additional variation of the results presented in Figure 11, Figure 12, Figure 13 and Figure 14. Moreover, the samples used for the tests had relatively small dimensions. The size of samples is less important in the case of solid materials, but here composites were analysed, which do not have a uniform structure.

According to Figure 11 and Figure 12, the tested material SBR 1100 exhibited the biggest decrease of tensile strength due to accelerated ageing. This was one of the reasons why the authors decided to choose SBR 1050 for further research, despite the fact that the initial values of tensile strength and elongation at break were higher for SBR 1100 than SBR 1050.

Each material was tested on five samples and the obtained results were very close to each other, taking into account the characteristics of porous materials. Materials based on solid rubber tend to lose their elastic properties due to accelerated ageing. Composites behave sometimes differently. In some cases, accelerated ageing may even improve their strength properties. One of the reasons is a chemical reaction that occurs in the polyurethane adhesive due to high temperatures and UV radiation, which can lead to better properties of such materials, for example tensile strength and elongation at break for SBR 1000 and SBR 1050.

### 3.3. Permanent Deformation under Compression in Sub-Zero Temperatures

An influence of sub-zero temperatures on the permanent deformation in the compression tests performed on seven previously described materials was investigated. The tests were conducted based on ISO 815-2 [37].

**Figure 15:**
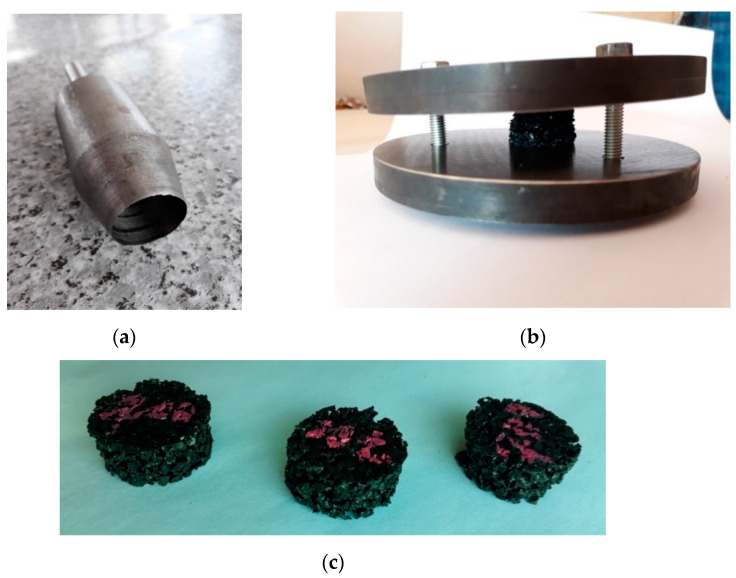
Views of: (**a**) Samples cutter; (**b**) Compressive plates; (**c**) Samples of elastomeric materials.

The following testing conditions were assumed (Figure 15):

- Cylindrical samples with the diameter of 25 mm and the height corresponding to the material thickness of ca. 10–12 mm;
- Number of samples: 3;
- Initial deformation: 10% of the initial height;
- Test temperature: −30 °C;
- Testing period: 168 h (7 days).

The samples were being cooled and compressed for 168 h, afterwards the pressure was removed and the height of the samples was measured after 10 s, 30 s, 1 min, 3 min, 10 min, 30 min and 2 h. After 2 h the samples were taken out of the climatic chamber.

The permanent deformation under compression (C) was calculated as:

(1) $$C=100\times \frac{h_{0}-h_{1}}{h_{0}-h_{s}},$$

where: h_0_—initial height of the sample, h_s_—height of the stand, h_1_—height of the sample after relaxation.

Analysis of the tests performed on seven materials based on SBR granulate with different densities (21 samples) leads to the following conclusions:

- For around 50% of the samples (11 out of 21) extensive deformation (140–180%) caused by shrinkage was observed at the temperature of −30 °C, which exceeded the initial compressive deformation of 100%;
- All samples had a tendency to return to their initial shape after relaxation;
- For 2 out of 21 samples the height after relaxation was bigger than the initial dimension;
- 5 out of 6 samples representing the materials with the highest shrinkage of 170–180% (SBR 850 and SBR 1100) did not return to shape after initial deformation of 100%, and thus did not regain their shape from before the applied initial deformation;
- In the case of four samples the permanent deformation was higher than 50% of the initial deformation, and in the case of 10 samples it was lower than 50%, which is around 5% of the real deformation.

Based on the conducted tests it can be stated that in severe freezing conditions (test deformation: 10% of the initial thickness, −30 °C, 168 h,) the materials kept the ability to return to their initial shape, in spite of the observed shrinkage. In more than half of the tested samples, from the initial deformation of 10%, less than 5% was permanent.

**Figure 16:**
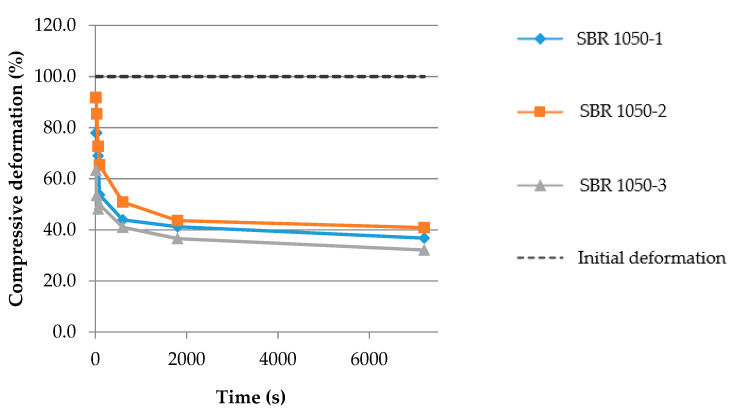
Samples’ deformation in the time domain, measured during relaxation after compression tests of the elastomeric material SBR 1050 at –30 °C; the deformation of 0% corresponds to the initial height of the sample before the test.

The tests indicated that in freezing conditions the most stable material was SBR 1050 (Figure 16). The tested samples exhibited similar behavior, did not undergo shrinkage and from the initial deformation of 10% only 3–4% was permanent.

### 3.4. Influence of Mineral Oil on the Dimensions of Samples

Although the role of the oil tests is diminished due to the development of rolling stock infrastructure and modern vehicles bought by railway carriers which do not loose oil anymore, there are still some old vehicles used by the Polish railway system. This is the reason why such tests should be performed when a new solution is to be introduced.

The tests of the influence of mineral oil on the properties of elastomeric materials were conducted based on ISO 1817 [38]. Mineral oil IRM 903 was used, which is a substance consisting of a strictly controlled mixture of two fractions of lubrication oils obtained through vacuum distillation of selected naphthenic oils (Gulf Coastal). This oil is described as a liquid that should cause large volume increases.

Six materials with various densities were tested (650, 700, 850, 1000, 1050, 1100 kg/m^3^) using the oar-shaped samples. After 7 days of full immersion in the oil with a temperature of 23 ± 2 °C and draining with tissue-paper, changes in dimensions were measured.

**Figure 17:**
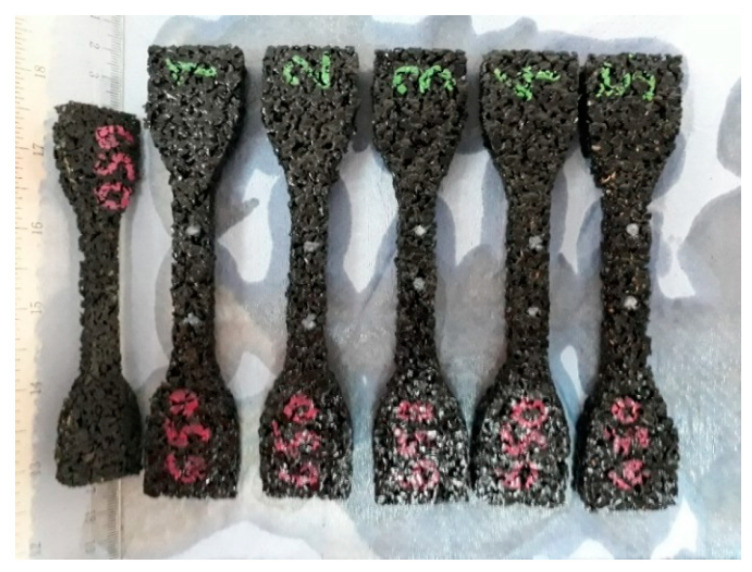
Change of length for sample SBR 650 after 7 days in oil.

The tests proved that:

- The samples lost some of their strength after they were taken out of oil and drained;
- Length of the samples increased (Figure 17, Table 5); samples with lower densities (650, 700, 850 kg/m^3^) increased their length by ca. 10% to 18%; samples with higher densities (1000, 1050, 1100 kg/m^3^) exhibited lower swelling and increased their length by ca. 6% to 8%.

### 3.5. Resistance in Contact with Ballast Grains

The resistance test of the elastic covering of rail dampers submitted to the impact of sharp edged ballast grains was realized on prototype samples in real scale, whose shape and dimensions were adjusted to Vignole rails: 49E1 and 60E1 (commonly used in Poland). Based on the results of previously described analyses and the material preselection in regard to their operational durability, the resistance test was conducted on the elastomeric material with a high density, SBR 1050.

The contact with sharp edged ballast grains was simulated during the test by the geometric ballast plate (GBP) imposing dynamic pressure with a frequency of 5 Hz. Such plates are used in the tests of various elastic elements of track structures, for example under sleeper pads (USP) [26,27] which are tested according to EN 16730 [39] or under ballast mats (UBM) [28] which are tested according to EN 17282 [40].

**Figure 18:**
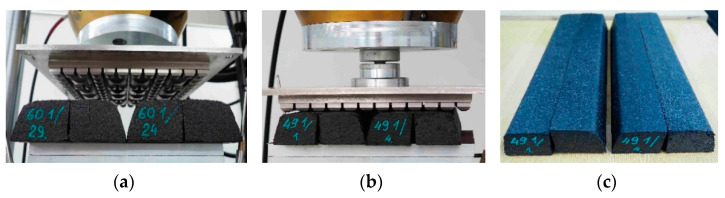
Prototype rail dampers with the elastic cover made of rubber granulate SBR 1050 during the test of resistance in contact with ballast grains: (**a**) Rail dampers adjusted to the Vignole rail 60E1 before the test; (**b**) Rail dampers adjusted to the Vignole rail 49E1 during the test; (**c**) Rail dampers adjusted to the Vignole rail 49E1 after the test—no visible damages or permanent deformations.

Before the test, the samples were visually inspected and no damage was noticed on their surfaces. After 100 thousand cycles of dynamic loading were applied to the rail dampers (four samples for each rail type; see Figure 18a,b) using the GBP, no damage or permanent deformations of the elastic cover were noticed (Figure 18c).

### 3.6. Conclusions from the Experimental Verification of the Operational Durability

Results of the performed tests lead to the following conclusions:

- The best results from the point of view of operational durability were exhibited by the samples of SBR granulate with high densities, that is, SBR 1000, SBR 1050 and SBR 1100;
- The worst results were obtained for the samples with lower densities, that is, SBR 850, SBR 750, SBR 700 and SBR 650;
- In severe freezing conditions the materials kept the ability to return to their initial shape, in spite of the observed shrinkage. In more than half of the tested samples, from the initial deformation of 10%, less than 5% was permanent. The most stable material was SBR 1050;
- Samples with lower densities (SBR 850, SBR 700 and SBR 650) exhibited significant swelling after contact with oil (the volume increase above 10%), which is an unacceptable result;
- In the resistance test conducted on the elastomeric material with a high density (SBR 1050), no damages or permanent deformations of the elastic cover were noticed after 100 thousand cycles of dynamic loading;
- The obtained results proved that the analysed materials did not lose their structural continuity under tension or compression; the determined elongation at break for porous SBR-based samples with densities over 850 kg/m^3^ was higher than 80% and there were no damages on the outer surface of prototypical rail dampers with a density of 1050 kg/m^3^ after 100 thousand load cycles.

## 4. Conclusions

Prototype rail dampers can be applied as elements that protect people and the surrounding environment against the negative influence of railway traffic noise. Such products have never been used in Polish railways before, but based on foreign applications (where primarily rail dampers with elastic polyurethane coverings have been implemented) they exhibit great potential for increasing the effectiveness of noise level suppression compared to traditional methods.

In the present paper, the results of a series of laboratory tests performed on seven different elastomeric materials made of SBR granulate were presented. The tested materials differed in density as follows: 1100, 1050, 1000, 850, 750, 700 and 650 kg/m^3^. It was proven that the rubber granulate SBR, produced from recycled waste tires, can be used as an elastic cover of the steel insert in rail dampers, provided that the material density is not lower than 1000 kg/m^3^. Samples of the materials with high densities exhibited good static and dynamic elastic characteristics and had sufficient operational durability.

The experimental identification of static and dynamic elastic characteristics revealed that the variations of static and dynamic properties were small in the case of SBR samples with densities of 1000 kg/m^3^ or higher, and much larger with lower densities. Tensile strength and elongation at break decreased significantly for SBR samples with densities below 1000 kg/m^3^. Application of a material with a minimum density of 1000 kg/m^3^ ensured a tensile strength over 2 MPa and an elongation at break over 80%.

The best results from the point of view of operational durability were exhibited by the samples of SBR granulate with high densities, that is, SBR 1000, SBR 1050 and SBR 1100. Samples with lower densities (SBR 850, SBR 700 and SBR 650) had much worse properties; additionally, they exhibited significant swelling after contact with oil (the volume increase above 10%), which is an unacceptable result. In severe freezing conditions the materials retained the ability to return to their initial shape in spite of the observed shrinkage. In more than half of the tested samples, less than 5% of the initial deformation of 10% was permanent. The most stable material was SBR 1050.

## Figures and Tables

**Table 1:** Static bedding moduli of the elastic cover of prototype rail dampers obtained for seven densities of SBR granulate (650–1100 kg/m^3^).

Assessed Loads Range (N/mm^2^)	Density (kg/m^3^)						
	1100	1050	1000	850	750	700	650
	Static Bedding Modulus (N/mm^3^)						
0.005 ÷ 0.02	0.125	0.126	0.130	0.129	0.115	0.104	0.091
0.01 ÷ 0.04	0.230	0.225	0.231	0.218	0.173	0.148	0.121
0.02 ÷ 0.05	0.347	0.328	0.337	0.307	0.214	0.177	0.136
0.02 ÷ 0.07	0.438	0.407	0.426	0.369	0.235	0.189	0.143

**Table 2:** Dynamic bedding moduli of the elastic cover of prototype rail dampers obtained for seven densities of SBR granulate (650–1100 kg/m^3^) within the applied and assessed loads range of 0.010 ÷ 0.040 N/mm^2^.

Load Frequency *f* (Hz)	Density (kg/m^3^)						
	1100	1050	1000	850	750	700	650
	Dynamic Bedding Modulus (N/mm^3^)						
1	0.287	0.275	0.219	0.207	0.137	0.131	0.126
2	0.365	0.347	0.365	0.343	0.260	0.221	0.176
3	0.380	0.367	0.377	0.357	0.269	0.228	0.182
5	0.394	0.393	0.390	0.376	0.278	0.238	0.188
10	0.420	0.402	0.422	0.401	0.295	0.252	0.200
15	0.430	0.408	0.438	0.402	0.306	0.257	0.206
20	0.457	0.435	0.448	0.418	0.314	0.274	0.217

**Table 3:** Variance analysis for the tensile strength (f_t_): SS—sum of squares, MS—mean squares, df—degree of freedom, F—test with a significance level (p) 0.05.

Factor	df	f_t_ (MPa)			
		SS	MS	F	p
Density	65.8293	5	13.1659	447.945	0.000000
Conditions	1.3380	1	1.3380	45.524	0.000000
Density*Conditions	3.2602	5	0.6520	22.184	0.000000
Sampling error	1.4108	48	0.0294		
General	65.8293	5	13.1659	447.945	0.000000

**Table 4:** Variance analysis for the elongation at break: SS—sum of squares, MS—mean squares, df—degree of freedom, F—test with a significance level (p) 0.05.

Factor	df	Elongation at Break (%)			
		SS	MS	F	p
Density	5	18,058.4	3611.7	91.798	0.000000
Conditions	1	660.4	660.4	16.786	0.000160
Density*Conditions	5	1378.2	275.6	7.006	0.000055
Sampling error	48	1888.5	39.3		
General	59	21,985.5			

**Table 5:** Dimension changes of elastomeric materials after immersion in oil IRM 903.

**Density (kg/m^3^)**	**650**			**700**			**850**		
	**Change of Length**			**Change of Length**			**Change of Length**		
**No.**	**l_0_** **(mm)**	**l_k_ (mm)**	**Δl** ** (%)**	**l_0_ (mm)**	**l_k_ (mm)**	**Δl** ** (%)**	**l_0_ (mm)**	**l_k_ (mm)**	**Δl** ** (%)**
1	12.9	15.2	17.8	12.8	15.1	18.0	12.9	14.2	10.1
2	13.0	15.4	18.5	12.9	15.0	16.3	13.0	14.4	10.8
3	13.1	15.4	17.6	12.9	15.0	16.3	12.9	14.4	11.6
4	12.9	15.1	17.1	13.0	15.1	16.2	13.0	14.5	11.5
5	12.9	15.4	19.4	12.9	15.1	17.1	13.0	14.1	8.5
**Mean** **value**	13.0	15.3	18.1	12.9	15.1	16.7	13.0	14.3	10.5
Standard deviation	0.1	0.1	0.9	0.1	0.1	0.8	0.1	0.2	1.3
**Density (kg/m^3^)**	**1000**			**1050**			**1100**		
	**Change of Length**			**Change of Length**			**Change of Length**		
**No.**	**l_0_** **(mm)**	**l_k_ (mm)**	**Δl** ** (%)**	**l_0_ (mm)**	**l_k_ (mm)**	**Δl** ** (%)**	**l_0_ (mm)**	**l_k_ (mm)**	**Δl** ** (%)**
1	12.0	12.7	5.8	13.0	14.3	10.0	13.0	13.8	6.2
2	12.2	12.7	4.1	13.0	13.9	6.9	12.9	13.8	7.0
3	12.2	13.0	6.6	13.1	14.2	8.4	12.8	13.4	4.7
4	12.3	13.0	5.7	12.9	13.8	7.0	12.9	13.7	6.2
5	12.2	13.0	6.6	13.0	14.0	7.7	13.0	13.7	5.4
**Mean** **value**	12.2	12.9	5.7	13.0	14.0	8.0	12.9	13.7	5.9
Standard deviation	0.1	0.2	1.0	0.1	0.2	1.3	0.1	0.2	0.9

## Data Availability

The data presented in this study are available on request from the corresponding author. The data are not publicly available due to the restrictions of the realized project and the authors’ will to patent some of the invented solutions.
